# Supplementary figures and images for: Untargeted metabolomic analyses of fermented unpolished black rice with melanogenesis inhibition activity
Source: PeerJ. 2025 Jun 4;13:e19533. doi: 10.7717/peerj.19533 (PMC12145086; doi:10.7717/peerj.19533)

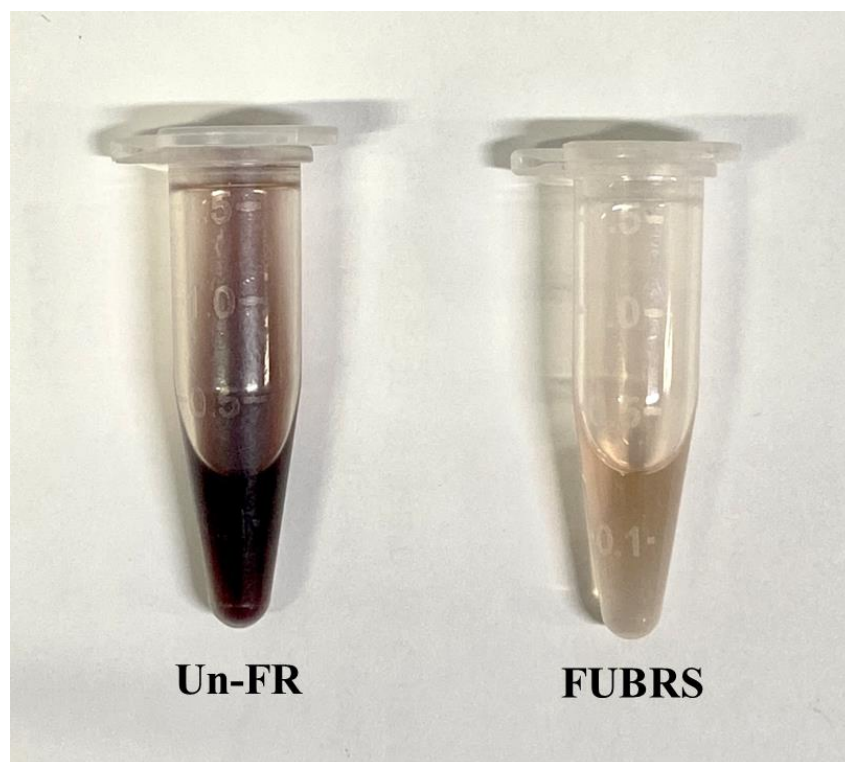

**Supplemental Figure S1: Characteristics of the Un-FR and FUBRS**

Supplement: Supplemental Information 1 [file peerj-13-19533-s001.pdf]
