# Supplementary material for: Untargeted metabolomic analyses of fermented unpolished black rice with melanogenesis inhibition activity
Source: PeerJ. 2025 Jun 4;13:e19533. doi: 10.7717/peerj.19533 (PMC12145086; doi:10.7717/peerj.19533)
Supplement: Supplemental Information 2 — Note: R2X, R2Y represent the model interpretation rate; Q2 indicates the model predictive ability. The closer R2Y and Q2 are to 1 the more stable and reliable is the model. [file peerj-13-19533-s002.pdf]

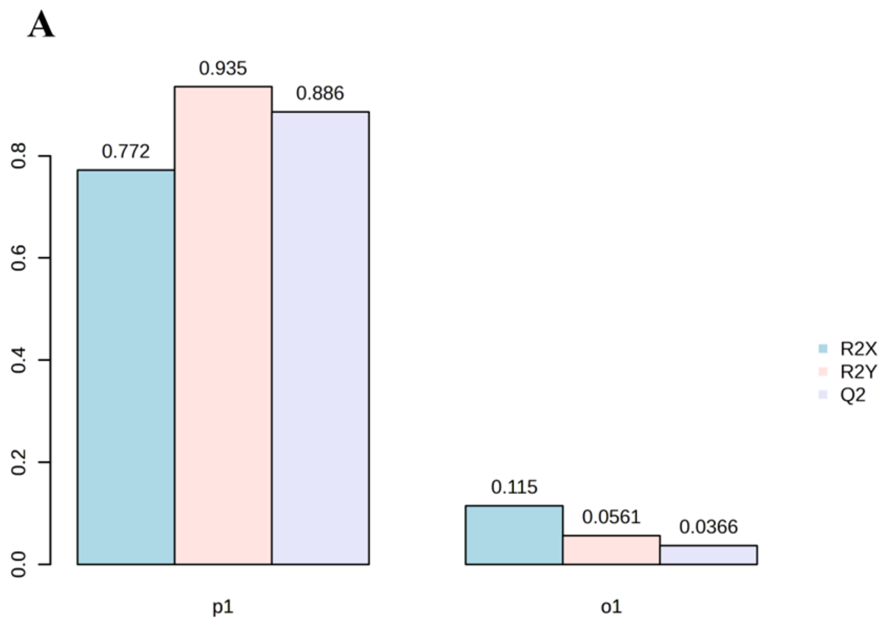

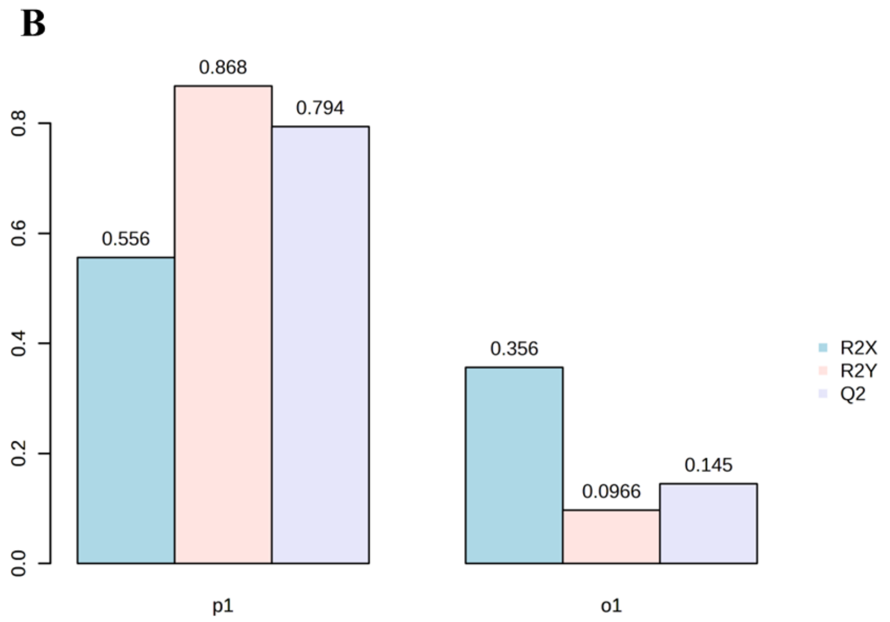

Supplemental Figure S2: The OPLS-DA permutation of (A) metabolites and (B) fatty acids using the GC-MS data. Note: R2X, R2Y represent the model interpretation rate; Q2 indicates the model predictive ability. The closer R2Y and Q2 are to 1 the more stable and reliable is the model.
