# Supplementary material for: Untargeted metabolomic analyses of fermented unpolished black rice with melanogenesis inhibition activity
Source: PeerJ. 2025 Jun 4;13:e19533. doi: 10.7717/peerj.19533 (PMC12145086; doi:10.7717/peerj.19533)
Supplement: Supplemental Information 3 [file peerj-13-19533-s003.pdf]

Supplemental Table S1: The detailed data of metabolites from two-fold change (FC) in FUBRS relative to those in Un-FR with a *p*-value < 0.05

Metabolites	log2(FC)	Metabolites	log2(FC)
Succinic acid	7.6459	Linoleic acid	-2.2253
Arabinose	7.5636	Glucopyranose	-2.5019
Lactic Acid	6.464	Fumaric acid	-2.5975
Glucose	4.9181	Citric acid	-2.6925
Myo-Inositol	3.6773	Psicofuranose	-3.0464
Alanine	3.2443	Fructose	-3.2632
Mannobiose	3.2298	Protocatechoic acid	-3.6029
Phenylalanine	3.2179	Galactose oxime	-3.777
Glyceric acid	3.1223	Salicylic acid	-3.7993
Serine	3.1133	Threonine	UnSig
Lactose	2.8976	Vanillic acid	UnSig
3-Hydroxybenzoic acid	2.728	Xylitol	UnSig
Pyruvic acid	2.6469	γ-aminobutyric acid	UnSig
Melibiose	2.6051	Palmitic acid	UnSig
Tartronic acid	2.5758	Aspartic acid	UnSig
α-Hydroxyglutaric acid	2.5691	Valine	UnSig
Ribopyranose	2.5647	Stearic acid	UnSig
Trehalose	2.536	Myristic acid	UnSig
Levoglucosan	2.5232	Cellobiose	UnSig
Proline	2.5207	Palatinose	UnSig
3-Phenyllactic acid	2.5185	Galactopyranose	UnSig
Leucine	2.5149	Gentiobiose	UnSig
Oleic Acid	2.4136		
Phosphoric acid	2.3206		
Acetic acid	1.7656		

Note: UnSig represents not significantly different
