# Supplementary material for: Untargeted metabolomic analyses of fermented unpolished black rice with melanogenesis inhibition activity
Source: PeerJ. 2025 Jun 4;13:e19533. doi: 10.7717/peerj.19533 (PMC12145086; doi:10.7717/peerj.19533)
Supplement: Supplemental Information 4 [file peerj-13-19533-s004.pdf]

Supplemental Table S2: Active ingredients with melanogenesis and/or tyrosinase inhibition activity in FUBRS

Active ingredients	References
Succinic acid	(Zarubina et al. 2012)
Lactic Acid	(Chan et al. 2014; Lee et al. 2024)
Glucose	(Lee et al. 2020)
Myo-Inositol	(Jun et al. 2011)
Alanine	(Ishikawa et al. 2007)
Pyruvic acid	(Zhou & Sakamoto 2019)
Trehalose	(Bin et al. 2016; Ohtake & Wang 2011)
3-Phenyllactic acid	(Shin et al. 2023)
Leucine	(Ishikawa et al. 2007)
Oleic acid	(Ando et al. 2006)
Acetic acid	(Lee et al. 2024)
Linoleic acid	(Ando et al. 1999)
Salicylic acid	(Liu et al. 2021)
Vanillic acid	(Liu et al. 2019)
γ-aminobutyric acid	(Molagoda et al. 2021)
p-Hydroxybenzoic acid	(Park et al. 2012)

References

Ando H, Funasaka Y, Oka M, Ohashi A, Furumura M, Matsunaga J, Matsunaga N, Hearing VJ, and Ichihashi M. 1999. Possible involvement of proteolytic degradation of tyrosinase in the regulatory effect of fatty acids on melanogenesis. *J Lipid Res* 40:1312-1316.

Ando H, Wen ZM, Kim HY, Valencia JC, Costin GE, Watabe H, Yasumoto K, Niki Y, Kondoh H, Ichihashi M, and Hearing VJ. 2006. Intracellular composition of fatty acid affects the processing and function of tyrosinase through the ubiquitin-proteasome pathway. *Biochem J* 394:43-50. 10.1042/bj20051419

Bin B-H, Kim ST, Bhin J, Lee TR, and Cho E-G. 2016. The Development of Sugar-Based Anti-Melanogenic Agents. *International journal of molecular sciences* 17:583-583. 10.3390/ijms17040583

Chan C-F, Huang C-C, Lee M-Y, and Lin Y-S. 2014. Fermented Broth in Tyrosinase- and Melanogenesis Inhibition. *Molecules (Basel, Switzerland)* 19:13122-13135.

Ishikawa M, Kawase I, and Ishii F. 2007. Combination of Amino Acids Reduces Pigmentation in B16F0 Melanoma Cells. *Biological & pharmaceutical bulletin* 30:677-681. 10.1248/bpb.30.677

Jun H-j, Roh M, Kim HW, Houng S-J, Cho B, Yun EJ, Hossain MA, Lee H, Kim KH, and Lee S-J. 2011. Dual inhibitions of lemon balm (Melissa officinalis) ethanolic extract on melanogenesis in B16-F1 murine melanocytes: Inhibition of tyrosinase activity and its

gene expression. *Food Science and Biotechnology* 20:1051. 10.1007/s10068-011-0143-1

Lee HW, Lee YR, Park KM, Lee NK, and Paik HD. 2024. Antimelanogenic and Antioxidant Effects of Postbioics of Lactobacillus Strains in α-MSH-Induced B16F10 Melanoma Cells via CREB/MITF and MAPKs Signaling Pathway. *J Microbiol Biotechnol* 34:2279-2289. 10.4014/jmb.2408.08015

Lee SH, Bae IH, Lee ES, Kim HJ, Lee J, and Lee CS. 2020. Glucose Exerts an Anti-Melanogenic Effect by Indirect Inactivation of Tyrosinase in Melanocytes and a Human Skin Equivalent. *International journal of molecular sciences* 21. 10.3390/ijms21051736

Liu J, Jiang R, Zhou J, Xu X, Sun Z, Li J, Chen X, Li Z, Yan X, Zhao D, Zheng Z, and Sun L. 2021. Salicylic acid in ginseng root alleviates skin hyperpigmentation disorders by inhibiting melanogenesis and melanosome transport. *European Journal of Pharmacology* 910:174458. https://doi.org/10.1016/j.ejphar.2021.174458

Liu J, Xu X, Jiang R, Sun L, and Zhao D. 2019. Vanillic acid in Panax ginseng root extract inhibits melanogenesis in B16F10 cells via inhibition of the NO/PKG signaling pathway. *Biosci Biotechnol Biochem* 83:1205-1215. 10.1080/09168451.2019.1606694

Molagoda IMN, Kavinda MHD, Ryu HW, Choi YH, Jeong JW, Kang S, and Kim GY. 2021. Gamma-Aminobutyric Acid (GABA) Inhibits α-Melanocyte-Stimulating Hormone-

Induced Melanogenesis through GABA(A) and GABA(B) Receptors. *International journal of molecular sciences* 22. 10.3390/ijms22158257

Ohtake S, and Wang YJ. 2011. Trehalose: current use and future applications. *Journal of pharmaceutical sciences* 100 6:2020-2053.

Park SH, Oh TH, Kim SS, Kim JE, Lee SJ, and Lee NH. 2012. Constituents with tyrosinase inhibitory activities from branches of Ficus erecta var. sieboldii King. *J Enzyme Inhib Med Chem* 27:390-394. 10.3109/14756366.2011.593033

Shin M, Truong VL, Lee M, Kim D, Kim MS, Cho H, Jung YH, Yang J, Jeong WS, and Kim Y. 2023. Investigation of phenyllactic acid as a potent tyrosinase inhibitor produced by probiotics. *Curr Res Food Sci* 6:100413. 10.1016/j.crfs.2022.100413

Zarubina IV, Lukk MV, and Shabanov PD. 2012. Antihypoxic and antioxidant effects of exogenous succinic acid and aminothiol succinate-containing antihypoxants. *Bull Exp Biol Med* 153:336-339. 10.1007/s10517-012-1709-5

Zhou S, and Sakamoto K. 2019. Pyruvic acid/ethyl pyruvate inhibits melanogenesis in B16F10 melanoma cells through PI3K/AKT, GSK3β, and ROS-ERK signaling pathways. *Genes to Cells* 24:60-69.
